# Comment on “Contrastive pre-training and 3D convolution neural network for RNA and small molecule binding affinity prediction” by Sun and Gao

**DOI:** 10.1093/bioinformatics/btaf163

**Published:** 2025-06-20

**Authors:** Sowmya Ramaswamy Krishnan, Arijit Roy, M Michael Gromiha

**Affiliations:** Department of Biotechnology, Bhupat and Jyoti Mehta School of Biosciences, Indian Institute of Technology Madras, Chennai 600036, India; TCS Research (Life Sciences Division), Tata Consultancy Services, Hyderabad 500081, India; TCS Research (Life Sciences Division), Tata Consultancy Services, Hyderabad 500081, India; Department of Biotechnology, Bhupat and Jyoti Mehta School of Biosciences, Indian Institute of Technology Madras, Chennai 600036, India

Dear Editor,

In a recent article in *Bioinformatics*, Sun and Gao (2024) reported a structure-based method for predicting the RNA-ligand binding affinity using a pretrained CNN-based model (RLaffinity) based on the data in PDBbind database (Liu *et al.* 2017). The performance of the model has been reported to be significantly better than other existing methods in the literature. However, upon closer inspection of the dataset and model predictions, the method has several shortcomings such as: (i) mixing the data for RNA-ligand and DNA-ligand complexes in the datasets, (ii) utilizing double normalization to the predictive variable, (iii) inability of the model to generalize to unseen RNA-ligand complex structures, (iv) prediction performance is not better than existing methods, and (v) incorrect comparison with existing prediction methods in the domain. We have discussed these concerns in detail in the following sections with relevant examples and metrics.

## Presence of DNA-ligand complex structures from PDBbind among the training, validation, and test datasets

The canonical functional forms of DNA structures (B-form) and RNA structures (A-form) vary *in cellulo*, and hence, can have an impact on the structural features learned by the RLaffinity model for binding affinity prediction. Upon inspecting the dataset splits provided in the GitHub repository associated with the article (Sun and Gao 2024), we found multiple instances of DNA-ligand complexes as part of the RNA-ligand complex dataset used by the authors. For example, the 10 test datasets with 13 complexes each, were found to include 19 DNA-ligand complexes (1P96, 1QMS, 1QV4, 1QV8, 1R4E, 2MG8, 5W77, 6J2W, 6JJ0, 2JWQ, 408D, 1CVX, 1DB6, 1CVY, 316D, 1YKV, 209D, 1I9V, 407D), which amount to 26 test datapoints (20% of 130).

## MinMax transformation of output variable (pK_d_) affects generalization of model to unseen data

While the authors claim to have predicted the RNA-ligand binding affinity using the RLaffinity model (Sun and Gao 2024), the predicted value is a double-scaled version of the binding affinity (log-transformation + min-max normalization). MinMax normalized values are devoid of outlier effects, leading to artificially higher Pearson correlation coefficients, which underscore the true potential of the predictive model. The MinMax normalized pK_d_ values from 144 RNA-ligand complexes have been used to train, validate, and test the model. For the available RNA-ligand complex structures with pK_d_ values outside the range of normalization in PDBbind ([2.508, 10.958]) (Liu *et al.* 2017), the predictions from RLaffinity model may not generalize well, resulting in increased prediction error.

For example, previous studies have shown that theophylline-binding RNA aptamer can efficiently discriminate theophylline from caffeine by a 10 000-fold difference in affinity (Jenison *et al.* 1994). Consequently, the binding affinity of theophylline aptamer to caffeine has been reported to be 3500 μM (Jenison *et al.* 1994, Menichelli *et al.* 2022), which leads to the pK_d_ value of 2.455. Using the PDB structure 1EHT as the template, the theophylline aptamer-caffeine complex structure was prepared and passed as input to RLaffinity. The predicted min-max normalized log-scale K_d_ from RLaffinity is 0.426, which translates to a pK_d_ of 6.1077 and K_d_ of 0.78 μM. Comparing to experimental pK_d_ value (2.455), it showed a high absolute error of 3.652. This example highlights the pitfall of using min-max normalization of pK_d_ values for the predictive variable.

## Inability of RLaffinity model to generalize to unseen RNA-ligand complexes

We curated a dataset of 12 RNA-ligand complexes, which are not present among the 144 complexes used in the RLaffinity study (Sun and Gao 2024). The experimental binding affinity values (K_d_) of these complexes were taken from R-SIM (Krishnan *et al*. 2023) as reported in the associated publication in PDB. These 12 structures were tested using the source code implementation of the RLaffinity model (Sun and Gao 2024). Upon pre-processing of the dataset, only 7 PDB structures could be processed (7D7Y, 7D7X, 3SKZ, 6UC8, 3SLM, 6UC7, 6DN1) by the code due to RDKit errors in SDF/MOL2 parsing (6DN2, 6DN3, 7WIE, 7WIF, 7WII). The binding affinity values predicted by the RLaffinity model for these seven RNA-ligand complexes are provided in Table 1. We observed that while the experimental pK_d_ values of the seven complexes range from 3.42 to 8.09, the pK_d_ values obtained from back-transformation of the min-max normalized RLaffinity predictions are all in a narrow range of 5.03 to 5.90. Although the method shows a mean absolute error of 1.384, the correlation is very weak between experimental and predicted affinities with a Pearson’s Correlation Coefficient (PCC) of 0.571 and a Spearman’s Correlation Coefficient (SPCC) of 0.535. Notably, the complexes with high (6DN1 and 6UC8) and low pK_d_ values (7D7X and 3SLM) are predicted with high mean absolute error (MAE) (1.37 to 2.2). Further, as discussed above, RLaffinity is not able to correctly predict the binding affinity of RNA-ligand complexes, which have pK_d_ values beyond the range used for normalization.

**Table 1.** Binding affinity predictions obtained for 7 RNA-ligand complexes from RLaffinity (Sun and Gao 2024) and RSAPred (Krishnan *et al.* 2024).^a^

PDB ID	Ligand name	K_d_ (from literature)	pK_d_	RLaffinity prediction	RLaffinity pK_d_	RLaffinity MAE^c^	**RSAPred pK_d_** ^b^	RSAPred MAE
6DN1	BRX1151	8 nM	8.09	0.40	5.90	**2.19**	9.79	1.70
6UC8	8-amino guanine	36 nM	7.44	0.38	5.80	**1.64**	8.85	1.41
6UC7	N2-acetyl guanine	300 nM	6.52	0.39	5.86	0.66	7.39	0.87
3SKZ	Guanosine	5 µM	5.30	0.37	5.71	0.41	3.33	1.97
7D7Y	ATP	90.2 µM	4.04	0.29	5.03	0.99	2.4	1.64
7D7X	ADP	95.3 µM	4.02	0.34	5.39	**1.37**	2.58	1.44
3SLM	dGMP	379 µM	3.42	0.39	5.84	**2.42**	4.91	1.49

a All predicted values have been rounded off to two decimal places for clarity.

b The RSAPred riboswitch model was used for predictions, as all seven test datapoints are riboswitch-ligand interactions.

c The values in bold indicate the predictions from RLaffinity with MAE greater than 1.

## Prediction performance is not better than existing methods

The predictions from RLaffinity were also compared with the recent method, RSAPred (Krishnan *et al.* 2024), for RNA-ligand binding affinity prediction. It should be noted that these 12 RNA-ligand complexes were not part of either the training or test datasets used by RSAPred. From Table 1, it can be observed that the predictions from RSAPred range from 2.4 to 9.79, capturing the diversity in pK_d_ values collected from literature, compared to RLaffinity, which predicts within a narrow range of 5.03–5.90. Further, RSAPred showed a PCC and SPCC of 0.90 and 0.75, respectively, whereas RLaffinity has a PCC of 0.571 and SPCC of 0.535 on the unseen dataset of seven RNA-ligand complexes. This result shows that the performance of RLaffinity model is not better than the existing method, RSAPred.

## Incorrect comparison with existing methods

As part of model validation, RLaffinity (Sun and Gao 2024) was compared with three existing methods namely, Vina (Trott and Olson 2010), RF-Score (Ballester and Mitchell 2010), and RSAPred (Krishnan *et al.* 2024). In case of Vina and RF-Score, both methods were primarily trained to score protein-ligand complexes, and hence have been shown to be unreliable for comparison with RNA-specific methods by previous studies (Stefaniak and Bujnicki 2021, Agarwal *et al.* 2023).

On the other hand, the comparison with RSAPred (Krishnan *et al.* 2024) has been made without normalization of the predicted binding affinities. Hence, by comparing the correlation coefficients of absolute binding affinity predictions (pK_d_ from RSAPred) to relative predictions (MinMax normalized pK_d_ from RLaffinity), the authors have highlighted a significant improvement in model performance. Further, RSAPred is a suite of binding affinity prediction models specific to six RNA subtypes (Aptamers, miRNAs, Repeats, Ribosomal RNAs, Riboswitches, and Viral RNAs), which was not considered in predicting the binding affinity. Hence, the comparison is not appropriate with protein-ligand binding affinity methods as well as MinMax normalized pK_d_ from RLaffinity and pK_d_ from RSAPred.

## Conclusions

Our analysis emphasizes the necessity of considering several important factors to validate the performance of RNA-ligand binding affinity prediction methods. It includes (i) utilizing RNA-ligand complexes alone for developing a model and validation, (ii) avoiding min-max normalization of log-normalized binding affinity values for prediction, (iii) testing the ability of the model to generalize well to unseen RNA-ligand interactions, (iv) improving the performance of the method, and (v) providing proper comparison with existing methods in the domain.

Conflict of interest: S.R.K. and A.R. are affiliated to Tata Consultancy Services Limited at the time of submission.

## Data Availability

All data shown in the manuscript is available from PDB database and RSAPred web server.
